# Pharmaceutically treated anxiety but not depression prior to cancer diagnosis predicts the onset of cardiovascular disease among breast cancer survivors

**DOI:** 10.1007/s10549-017-4387-1

**Published:** 2017-07-17

**Authors:** Dounya Schoormans, Lonneke van de Poll-Franse, Pauline Vissers, Myrthe P. P. van Herk-Sukel, Susanne S. Pedersen, Nina Rottmann, Trine Horsbøl, Susanne Dalton, Johan Denollet

**Affiliations:** 1 Department of Medical and Clinical Psychology, CoRPS - Center of Research on Psychology in Somatic Diseases, Tilburg University, Warandelaan 2, 5000 LE Tilburg, The Netherlands; 2 Comprehensive Cancer Organization Netherlands Eindhoven, Eindhoven, The Netherlands; 3 Department of Psychology, University of Southern Denmark, Odense, Denmark; 4 Division of Psychosocial Research and Epidemiology, Netherlands Cancer Institute, Amsterdam, The Netherlands; 5 PHARMO Institute for Drug Outcomes Research, Utrecht, The Netherlands; 6 Department of Cardiology, Odense University Hospital, Odense, Denmark; 7 Department of Public Health, National Research Center for Cancer Rehabilitation, University of Southern Denmark, Odense, Denmark; 8 Survivorship Unit, Danish Cancer Society Research Center, Copenhagen, Denmark

**Keywords:** Breast cancer survivors, Cardiovascular disease, Anxiety, Depression, Cardiotoxicity

## Abstract

**Purpose:**

To examine the associations between pharmaceutically treated anxiety and depression present in the year prior to breast cancer diagnosis and the risk of incident cardiovascular disease (CVD), while controlling for traditional cardiovascular risk factors and clinical characteristics in a population-based observational study.

**Methods:**

Adult 1-year breast cancer survivors (*n* = 7227), diagnosed between 01-01-1999 and 12-31-2010, with no history of CVD, were selected from the Netherlands Cancer Registry. Drug dispensing data were derived from the PHARMO Database Network and used as proxy for CVD, anxiety, and depression. By multivariable Cox regression analysis, we examined the risk associated with pharmaceutically treated anxiety and depression for developing CVD after cancer diagnosis, adjusting for age, pharmaceutically treated hypertension, hypercholesterolemia, and diabetes mellitus in the year prior to cancer diagnosis, tumor stage, and cancer treatment.

**Results:**

During the 13-year follow-up period, 193 (3%) breast cancer survivors developed CVD. Women pharmaceutically treated for anxiety in the year prior to their cancer diagnosis had a 48% increased hazard for CVD [HR = 1.48; 95% CI 1.05–1.08] after full adjustment. This association was restricted to breast cancer survivors who were 65 years or younger. Depression was not associated with CVD risk [HR = 0.89; 95% CI 0.52–1.53]. Older age [HR = 1.06; 95% CI 1.05–1.08], hypertension [HR = 1.80; 95% CI 1.32–2.46], and hypercholesterolemia [HR = 1.63; 95% CI 1.15–2.33] were associated with an increased hazard for incident CVD, whereas hormone therapy [HR = 0.59; 95% CI 0.42–0.83] was protective.

**Conclusions:**

Anxiety present in the year prior to breast cancer diagnosis increases the risk of incident CVD in 1-year breast cancer survivors, after adjustment for depression, traditional cardiovascular risk factors, and clinical characteristics.

## Introduction

Cardiovascular disease (CVD) is a common comorbidity in breast cancer survivors [1, 2] and estimated to be responsible for many non-cancer-related mortalities [1]. This is partly due to aging, yet also a consequence of received treatment. The cardiotoxicity of cancer treatment can lead to the development of a wide range of CVDs, such as arrhythmias, heart failure, and valvular heart disease [3, 4]. Furthermore, there are similar underlying risk factors for both breast cancer and CVD, such as obesity and physical inactivity [5–7].

Incidence rates of CVD in breast cancer survivors vary depending on the type of treatment and dosage [8]. Even in individuals who all received the exact same treatment and dosage not all develop CVD [9]. Hence, additional factors, other than cardiotoxic treatment, are involved and may include traditional cardiovascular risk factors, such as hypertension or diabetes mellitus, as they increase the risk of CVD in breast cancer survivors [7, 10, 11].

Psychological factors, such as anxiety and depression, may also increase the risk of CVD in breast cancer survivors. Several studies have shown that depression and anxiety are predictive for the development and progression of CVD in non-cancer patients [12–16]. A meta-analysis based on 20 studies examining the predictive value of anxiety for the incidence of CVD in healthy individuals concluded that anxious patients had a 26% increased risk of developing CVD and a 48% increased risk of cardiac death [12]. Furthermore, depression is a risk factor for recurrent cardiac events, heart failure [13, 14], major adverse cardiac events [14, 15], and cardiac death [13]. Hence, the European Society of Cardiology included psychosocial risk factors, including anxiety and depression, in the Guidelines on Cardiovascular Disease Prevention in Clinical Practice in 2012 [11]. Moreover, in a previous study among middle-aged healthy women (similar in age to breast cancer survivors), anxiety was found to be predictive of cardiac mortality [16].

CVD is understudied, underdiagnosed, and undertreated among women, yet we know that there are sex differences in the pathophysiology of CVD [17]. Simultaneously, levels of anxiety and depression are more prominent among female cancer survivors than in males [18]. Nonetheless, to our knowledge, the association between anxiety and depression with incident CVD among female breast cancer survivors has never been studied before. The aim of this study was therefore to examine the associations between anxiety and depression present in the year prior to a breast cancer diagnosis and the risk of incident CVD in 1-year breast cancer survivors, while controlling for age, traditional cardiovascular risk factors, tumor stage, and cancer treatment (i.e., chemo-, radio-, and hormone therapy). We chose to look at 1-year survivors as cancer treatment is generally finished within the first year. In addition, we explored whether the associations between anxiety and depression with incident CVD risk differed by age, traditional cardiovascular risk factors, or cancer treatment.

## Methods

### Procedure and participants

Data from the Southern Region of the Netherlands Cancer Registry (NCR) were used in this observational cohort study. The NCR registers cancer diagnosis, stage, and primary cancer treatment for all newly diagnosed cancer patients and is maintained by the Netherlands Comprehensive Cancer Organization [19]. The Southern Region of the NCR covers an area of 2.4 million inhabitants [19]. For this study, the NCR was linked to data from the PHARMO Database Network for cancer patients diagnosed from 1998 onwards, and a detailed description of this linkage is found elsewhere [20]. PHARMO is a large, population-based network of electronic healthcare databases and combines data from general practices, pharmacies, and hospitals which are linked on patient-level though validated algorithms. In this study, the out-patient pharmacy database comprising healthcare products prescribed by the general practitioner or specialist was used. Dispensing records used included information on product type and date. Drug dispensings are coded according to the international Anatomical Therapeutic Chemical (ATC) classification system [21].

Female adult breast cancer patients diagnosed between 01-01-1999 and 12-31-2010 were selected from the NCR. To obtain information on survival status and date of death, the NCR was linked to the municipal Personal Records database. As anonymous observational patient information was used, this study does not fall under the Medical Research Involving Human Subjects Act in the Netherlands; therefore, this study was exempted from medical ethics review and no informed consent was required. This study was performed in agreement with the Declaration of Helsinki.

Breast cancer survivors who had a history of CVD medication use (see the definition of CVD in the next section) in the 12 months prior to their cancer diagnosis were excluded, as our aim was to examine the risk of incident CVD following cancer diagnosis. In addition, to exclude the effect of detecting CVD due to increased clinical checkups and the direct and sometimes reversible effects of receiving cancer treatment, breast cancer survivors who developed CVD in the first year after diagnosis were also excluded. Follow-up for a diagnosis of CVD began 12 months after the cancer diagnosis (which was set as the index date), as primary cancer treatment is generally finalized within the first year. Hence, we excluded those who died or were lost to follow-up during the first year. Follow-up time was measured until onset of CVD, death, loss to follow-up, or until the end of the study period (31-12-2010), whichever occurred first.

### Measurements

#### Cardiovascular disease

CVD was defined as having at least two drug dispenses of *cardiac therapeutics* (i.e., ATC code C01) at unique dates within 6 months. Survivors who dispensed two cardiac drugs with less than 15 days in between were classified as having CVD only when they had three cardiac dispenses at unique dates in a 6-month period. We used a strict definition based solely on using cardiac therapeutics (ATC = C01) to avoid false classifications of CVD. Therefore, usage of CVD-related drugs such as diuretics (C03) or beta-blockers was insufficient to be classified as having CVD, as these drugs have a broad treatment range including non-CVD indications.

#### Psychological factors—anxiety and depression

Drug dispense information for anxiety disorders (ATC = N05B) and depression (ATC = N06A) during the 12 months prior to the cancer diagnosis was included. Patients with one or more drug dispensings were categorized as anxious or depressed (yes/no).

#### Traditional cardiovascular risk factors

Drug dispense information on the traditional cardiovascular risk factors including hypertension (ATC = C02, C03A, CO3B (except C03C), C07, C08, C09 (except C09X)), hypercholesterolemia (ATC = C10), and diabetes mellitus (ATC = A10) during the 12 months prior to the cancer diagnosis was captured [22]. Having one or more drug dispensings for hypertension, hypercholesterolemia, and diabetes mellitus in the 12 months prior to the cancer diagnosis was categorized as having traditional cardiovascular risk factors (yes/no). We opted for the inclusion of these traditional cardiovascular risk factors already present prior to cancer diagnosis, as cardiotoxic treatment is known to increase the risk of developing these traditional cardiovascular risk factors, which can then be seen as a precursor of cancer treatment-induced CVD itself.

#### Age and clinical characteristics

Survivors’ age and clinical information on tumor stage and treatment (i.e., having received chemo-, radiation-, or hormone therapy (yes/no)) were obtained from the NCR.

### Statistical analyses

Differences in patient characteristics (i.e., age, psychological factors, traditional cardiovascular risk factors, and clinical characteristics) between breast cancer survivors with and without incident CVD were analyzed using ANOVA, the *χ* ^2^ test, or Student’s *t* test for independent samples as appropriate.

The associations between pharmaceutically treated anxiety and depression with incident CVD risk were examined separately and simultaneously using multivariable Cox regression analyses. Analyses included covariates which were entered in separate steps. First, we adjusted for age (continuous) as a potential confounder. Second, traditional cardiovascular risk factors (i.e., hypertension, hypercholesterolemia, and diabetes mellitus) were added to the model. Finally, clinical information on tumor stage, chemo-, radio-, and hormone therapy was entered, which were considered possible explanatory variables. Assumptions underlying the multivariable Cox regression analysis were met (e.g., visual inspection of the KM curve allowed confirmation of the Cox proportional hazard assumptions).

We tested effect modifications for age, traditional cardiovascular risk factors, and cancer treatment (chemo-, radio-, and hormone therapy) by adding interaction terms (i.e., depression/anxiety*age/traditional cardiovascular risk/cancer treatment) to the fully adjusted model. We examined whether the effect of anxiety and depression on CVD risk differed by age (≤65 vs >65 years at the time of cancer diagnosis), traditional cardiovascular risk factors, chemotherapy, radiation, or hormonal treatment. Missing data were handled in previous steps and described elsewhere [20]. All statistical tests were two-sided with alpha set at 5%. We chose not to use a more stringent alpha level since this is the first study relating both pharmaceutically treated anxiety and depression to CVD risk in a sample of breast cancer survivors, and hence we wanted to avoid making a type 2 error. All analyses were performed using SPSS version 22.0.

## Results

### Patient characteristics

Of the 7889 eligible breast cancer survivors, 515 were excluded as they received prescribed CVD medications in the 12 months before or after their cancer diagnosis, and 147 were excluded as they were deceased or lost to follow-up in the first year after cancer diagnosis. After exclusion, 7227 1-year breast cancer survivors were included in statistical analyses.

The 1-year breast cancer survivors who developed CVD differed from those who did not (Table 1)—that is—they had a 1-year shorter follow-up period, had higher mortality rates, used more often drugs for anxiety and traditional cardiovascular risk factors (i.e., hypertension, hypercholesterolemia, diabetes mellitus) in the year prior to their cancer diagnosis, and were treated less often with chemo-, radio-, and hormone therapy (all *p*’s <0.05).

**Table 1:** Patient characteristics of 7227 1-year breast cancer survivors stratified by CVD status

	CVD (*n* = 193)	No CVD (*n* = 7034)
Follow-up^b^ time in median years (range)	3 (0–13)	4 (0–13)*
Deceased	53 (28)	1077 (15)*
Demographics		
Age in median years (range)	70 (46–91)	60 (23–102)*
Psychological factors		
Anxiety	45 (23)	976 (14)*
Depression	15 (8)	553 (8)
Traditional CVD risk factors^a^	118 (61)	2214 (32)*
Hypertension	106 (55)	1892 (27)*
Hypercholesterolemia	49 (25)	810 (12)*
Diabetes mellitus	21 (11)	398 (6)*
Clinical characteristics		
Tumor stage		
1	93 (49)	3119 (45)
2	80 (42)	2819 (41)
3	14 (7)	761 (11)
4	3 (2)	223 (3)
Cancer treatment		
Surgery	184 (95)	6677 (95)
Chemotherapy	29 (15)	2661 (38)*
Radiation	123 (64)	5042 (72)*
Hormone therapy	72 (37)	3392 (48)*

Information is provided in numbers (*n*) with percentages for categorical variables, whereas follow-up time and age are presented in median years (range). CVD = cardiovascular disease. Psychological and traditional CVD risk factors = being pharmaceutically treated in the 12 months prior to breast cancer diagnosis

^a^Being pharmaceutically treated for at least one of the traditional cardiovascular risk factors (hypertension, hypercholesterolemia, diabetes mellitus) during the 12 months prior to cancer diagnosis, yes/no

^b^Follow-up for a diagnosis of CVD began 12 months after cancer diagnosis, as primary cancer treatment is generally finalized within the first year

*Significant difference (*p* < 0.05) between those with and without CVD

### Associations with CVD risk

Analyzing the associations between anxiety and depression present in the year prior to cancer diagnosis with CVD risk separately (data not shown) showed that anxiety in the year prior to cancer diagnosis was associated with an increased risk for incident CVD in all models: age-adjusted [hazard ratio (HR) = 1.58; 95% confidence interval (95% CI) 1.13–2.20], partially adjusted (adjusted for age and CVD risk factors) [HR = 1.45; 95% CI 1.04–2.03], and fully adjusted model [HR = 1.46; 95% CI 1.04–2.04). Surprisingly, depression was not significantly associated with CVD risk in any of the models (data not shown).

When adding both anxiety and depression simultaneously to the model (Table 2), anxiety remained associated with an increased risk for incident CVD [HR = 1.60; 95% CI 1.13–2.25], while controlling for depression. The adjustment for traditional cardiovascular risk factors slightly attenuated the effect of anxiety but remained significant [HR = 1.47; 95% CI 1.04–2.07]. This did not change after adding information on tumor stage and treatment to the model [HR = 1.48; 95% CI 1.05–2.08]. Hence, women who were anxious in the year prior to their cancer diagnosis had a 48% increased CVD hazard after adjustment for depression, traditional cardiovascular risk factors, and clinical factors. Depression was not associated with incident CVD in any of the models (Table 2).

**Table 2:** Associations between pharmaceutically treated anxiety and depression with incident CVD risk after breast cancer diagnosis

	Age-adjusted model	Partially adjusted model	Fully adjusted model
	HR (95% CI)	HR (95% CI)	HR (95% CI)
Psychological factors			
Anxiety	1.60 (1.13–2.25)*	1.47 (1.04–2.07)*	1.48 (1.05–2.08)*
Depression	0.91 (0.53–1.57)	0.90 (0.53–1.55)	0.89 (0.52–1.53)
Demographics			
Age (continuous)	1.07 (1.06–1.08)*	1.06 (1.05–1.08)*	1.06 (1.05–1.08)*
Traditional CVD risk factors			
Hypertension	–	1.77 (1.29–2.42)*	1.80 (1.32–2.46)*
Hypercholesterolemia	–	1.77 (1.29–2.42)*	1.63 (1.15–2.33)*
Diabetes mellitus	–	1.02 (0.63–1.64)	1.05 (0.65–1.69)
Clinical characteristics			
Tumor stage			
Stage I (reference)	–	–	–
Stage II	–	–	1.14 (0.81–1.61)
Stage III	–	–	0.97 (0.52–1.80)
Stage IV	–	–	0.93 (0.29–3.05)
Cancer treatment			
Chemotherapy	–	–	1.05 (0.65–1.69)
Radiation	–	–	0.78 (0.57–1.06)
Hormone treatment	–	–	0.59 (0.42–0.83)*

Information is provided in hazard ratios (HR) with 95% confidence intervals (95% CI). Partially adjusted = adjusted for age and the traditional cardiovascular risk factors (i.e., hypertension, hypercholesterolemia, and diabetes mellitus) present in the 12 months prior to cancer diagnosis; fully adjusted = adjusted for traditional cardiovascular risk factors and clinical information (tumor stage and treatment information, that is chemotherapy, radiation, and hormone treatment). Psychological and traditional CVD risk factors = being pharmaceutically treated in the 12 months prior to breast cancer diagnosis. *p < 0.05

Older age [HR = 1.06; 95% CI 1.05–1.08], taking medication for hypertension [HR = 1.80; 95% CI 1.32–2.46], and hypercholesterolemia [HR = 1.63; 95% CI 1.15–2.33] were associated with an increased hazard for incident CVD, whereas being treated with hormone therapy [HR = 0.59; 95% CI 0.42–0.83] was protective for CVD (Table 2). Taking medication for depression or diabetes mellitus, tumor stage, chemo- and radiotherapy were not associated with CVD risk (Table 2).

**Fig. 1:**
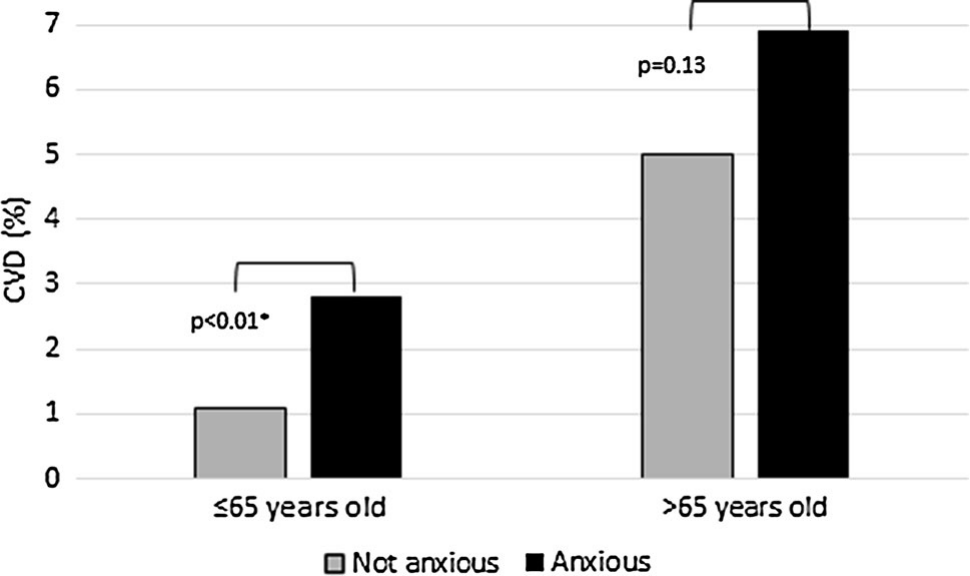
Association between pharmaceutically treated anxiety and incident CVD among younger (≤65 years) and older (>65) women (age at breast cancer diagnosis). Note CVD = cardiovascular disease, *p* = *p* value; *p < 0.05

There were no cancer treatment moderation effects for anxiety on CVD risk, nor were there significant interaction effects with traditional cardiovascular risk factors. There was an age-related significant interaction effect with anxiety [HR = 0.84; 95% CI 0.74–0.96], where there remained a significant main effect of anxiety [HR = 2.04; 95% CI 1.36–3.06] on CVD risk. Stratified analyses showed that the association was restricted to women who were ≤65 years at breast cancer diagnosis [HR = 2.29; 95% CI 1.31–4.02], as there was no increased CVD risk among older survivors [HR = 1.24; 95% CI 0.80–1.93] (Fig. 1).

## Discussion

This population-based observational study showed that pharmaceutical therapy for anxiety but not depression in the year prior to breast cancer diagnosis was associated with an increased hazard of incident CVD in 1-year breast cancer survivors. This relation remained significant after adjustment for depression, traditional cardiovascular risk factors, and clinical characteristics, and was restricted to younger breast cancer patients. Associations did not differ by type of cancer treatment or the presence of traditional cardiovascular risk factors in the year prior to breast cancer diagnosis.

The association between pharmaceutical treatment for anxiety present in the year prior to cancer diagnosis and the increased risk for CVD found in the current study is consistent with studies in healthy individuals, showing that anxiety predicts the onset of CVD [11, 12]. We focused on pharmaceutically treated anxiety and depression prior to breast cancer to examine whether anxiety and depression prior to the major life event of a breast cancer diagnosis are related to an increased risk of CVD. However, we know that being diagnosed with cancer and undergoing treatment is known to have a major impact on one’s life, as many cancer survivors experience feelings of anxiety or depression [23, 24]; hence, an additional proportion of breast cancer survivors may start to use pharmaceutical treatment for anxiety. We found a higher increased risk for anxiety in our study (48%) than a meta-analysis among healthy individuals did (26%) [12]. The increased risk of anxiety in our study remained statistically significant despite adjustment for hypertension, hypercholesterolemia, diabetes mellitus, tumor stage, and received chemo-, radio-, or hormone therapy. Hence, this association seems not to be driven by the presence of traditional cardiovascular risk factors, cancer stage, or cancer treatment. It is interesting that anxiety is only associated with an increased CVD risk in younger survivors (≤65 years). This is in line with previous research where psychological factors especially seem to play a role among younger individuals, whereas among older individuals aging—and likely physiological factors related to the aging and disease process—is suggested to drive the relationship with poor health outcomes [25]. Several behavioral and pathophysiological mechanisms have been suggested to underlie the associations between anxiety and increased CVD risk in non-cancer populations. Anxiety has, for example, been related to unhealthy lifestyle behaviors such as smoking and limited exercise [26]. Furthermore, the autonomous nervous system and hypothalamic–pituitary–adrenal axis known for their role in the pathogenesis of CVD [27, 28] have been suggested to be involved, as anxious individuals have a lower heart rate variability [29] and higher cortisol levels [30]. As medication use was used as a proxy for both anxiety and CVD, we cannot rule out that the association may partly be explained by a pharmacokinetic interaction between both drug types, although little is known about this association and the usage of anxiolytics is common among CVD populations [31, 32].

The lack of an association between depression and CVD risk was unexpected, as previous studies have demonstrated that depression is a risk factor for both incident CVD and CVD progression [11, 13–15]. This dissimilarity could be because often other studies have examined either depression or anxiety, and hence they are unable to disentangle the role of each of depression and anxiety separately and together. Alternatively, the result of the low prevalence of pharmaceutically treated depressed individuals in our study (8%) could play a role in us not confirming previous results. Using drug dispense information as an indicator of depression, we could be underestimating the true prevalence of depression, as a depression and its milder form depressive symptoms are often not treated with medication. Alternatively, depression may comprise different subtypes. It has been suggested that some manifestations of depression may partly reflect cardiac disease severity [33], and hence it may have distorted the strength of the previously found associations between depression and prognosis of CVD. Nevertheless, the fact that anxiety, but not depression, was significantly associated with increased CVD risk is in line with previous findings studying poor outcomes among CVD populations [34–36].

Surprisingly, there was no effect of chemo- or radiotherapy on CVD risk, despite the known cardiotoxicity of these treatments [3, 4]. This finding may be attributed to the lack of detailed information about type and dose of systemic therapy given. As not all chemotherapeutic agents are equally cardiotoxic, cardiotoxicity of chemo- and radiotherapy is dose dependent [3]. It is thus possible that grouping all chemotherapeutic agents irrespective of type and dosage will result in an underestimation of their true effect. Moreover, we found a protective effect of hormonal cancer treatment on CVD risk. A previous study also found that hormone treatment in breast cancer survivors can lower the risk of CVD, yet only several years after diagnosis, as it increases the risk of CVD in the first years after cancer diagnosis [37]. Previous studies have suggested that estrogen hormone treatment has favorable effects on lipoproteins, coronary arteriosclerosis, endothelial function, and arterial thrombosis [37]. Exploratory post hoc analyses, indeed, showed a protective effect of hormone treatment on incident CVD 6–10 years after cancer diagnosis in our sample, whereas there was no significant relation in the first 5 years after diagnosis or after ≥11 years.

A general limitation inherent to the observational study design is the lack of information on residual confounders. Furthermore, we used drug dispense information as a proxy for anxiety, depression, and CVD. We did not have additional information on medical diagnosis or patient-reported information on anxiety or depression. Nevertheless, algorithms based on pharmacy drugs are known to be more specific, yet less sensitive than medical diagnoses [38]. Also, we used a rather tight algorithm to define CVD, as this was based on a minimum of two C01 drug dispenses within 6 months, possibly leading to an underestimation of the incidence of CVD. It is also possible that we missed CVD patients who use other drugs, such as ACE inhibitors or beta-blockers but no C01 drug, although we expect this number to be small. In addition, as we were interested in incident CVD, breast cancer survivors with CVD in the year prior to their cancer diagnosis were excluded. Hence, we are looking at a subpopulation of 1-year breast cancer survivors.

Strengths of our study include the large population-based sample of breast cancer survivors and the usage of high-quality databases of the Netherlands Cancer Registry and PHARMO enabling a 13-year follow-up period. Additionally, this is to our knowledge the first study that examined the association between pharmaceutically treated anxiety and depression with incident CVD among breast cancer survivors. Furthermore, we estimated CVD risk 1 year after breast cancer diagnosis, striking a compromise between not starting too late and missing incident CVD due to ongoing cancer treatment or increased clinical checkups.

In conclusion, 1-year breast cancer survivors with pharmaceutically treated anxiety in the year prior to their cancer diagnosis had a 48% increased risk of incident CVD, after adjustment for depression, traditional cardiovascular risk factors, tumor stage, and cancer treatment. This increased risk seems to be limited to those breast cancer survivors who were 65 years or younger at cancer diagnosis. Depression was not related to an increased risk of incident CVD. Future studies unraveling these associations are warranted in order to provide the best optimal care for women treated for breast cancer.
